# Granular computing in mosaicing of images from capsule endoscopy

**DOI:** 10.1007/s11047-014-9477-y

**Published:** 2015-01-06

**Authors:** Lukasz Maciura, Jan G. Bazan

**Affiliations:** 1 Chair of Computer Science, University of Rzeszów, Pigonia 1, 35-310 Rzeszów, Poland; 2 Interdisciplinary Centre for Computational Modelling, University of Rzeszów, Pigonia 1, 35-310 Rzeszów, Poland

**Keywords:** Granular computing, Capsule endoscopy, Image registration, Image mosaic, Keypoints matching

## Abstract

This article introduces methods for modeling compound granules used in algorithms which could successfully construct a mosaic from the images coming from an endoscope capsule. In order to apply the algorithm, combined images must have a common area where the correspondence of points is determined. That allows to determine the transformation parameters to compensate movement of the capsule that occurs between moments when the mosaic images were acquired. The developed algorithm for images from the capsule endoscopy has proved to be faster and comparably accurate as commercial GDB-ICP algorithm.

## Introduction

**Fig. 1:**
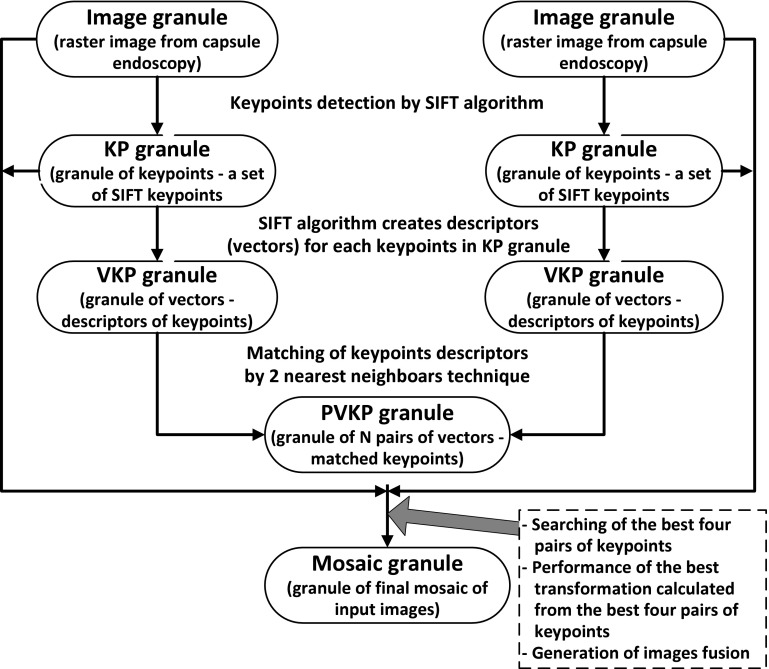
Granular computing for the mosaicing of two images

The study aimed at analyzing any available algorithms of image registration and mosaicing, developing selected algorithms and creating such an algorithm which could successfully construct a mosaic from the images coming from an endoscope capsule. Solving problems regarding image mosaicing requires techniques for combining information arriving from two or more images of different quality. The information included in these images is often inaccurate and incomplete. It results from the technical problems while acquiring images (e.g., noise). One of paradigms for dealing with such complex problems is *the granular computing* (see e.g., Doherty et al. 2006; Pedrycz et al. 2008). The basic notion for granular computing is *a granule* which is, in this work, understood as a piece of information or data. The granule may be assembled with other granules taking into account the possible relationships between these granules. In other words, granules can be treated as parts or elements of a more complex granules which finally can be treated as a solution of a given problem. Moreover, the structure of a complex granule can be hierarchical and each granule existing within such structure must represent well the data which it was established to represent. The granules may be processed by means of so called granular computing, by which the granules are created, altered, assembled or deleted, etc. The method suggested in this work uses the following granules and operations of granular computing. At the beginning we have two raster images coming from an endoscope capsule that we treat as two initial granules (see Sect. 2.1). Next, there is a transformation of both granules to the other two granules which we call *granules of keypoints* (KP granules—see Sect. 2.2). A separate KP granule is created for every image and it is a set of so called keypoints, i.e., the essential points of an image [the points found with the SIFT algorithm (Chen and Tian 2006; Lowe 2004)]. Both KP granules are further reduced by rejecting the unsure points (by means of thresholding of colour saturation), and leaving only these points which lie in the area of dominant edges and corners in an image (see Sect. 2.2). Every keypoint belonging to KP granules is enriched by the vector of qualities calculated on the basis of its surrounding. The vector characterizes the keypoint is used for further calculations. Thanks to it the KP granules are replaced by two VKP granules which are the collection of vectors calculated for all keypoints from equivalent KP granules. Next, a granule PVKP is created which includes $N$ pairs of vectors (in fact, pairs of keypoints) from the first and second image, under the condition that these are the points whose matching was top rated by a special algorithm which works on *a method of two closest neighbours*, but the special function of distance is constructed on the basis of vectors of previously set qualities for particular keypoints. Now, there are examined all four-element combinations of pairs of points from the granule PVKP created in the previous step (see Sect. 2.3). As a result of further calculations and additional tests (see Sects. 2.4, 2.6, 2.7) there comes out one granule of optimal four pairs which becomes *a mosaic granule* (because four pairs of keypoints is necessary for mosaicing). On the basis of a selected mosaic granule, finally there is performed transformation of pixels of both images into one final granule (see Sect. 2.9) that we interpret as a mosaic of images (a kind of a fusion between two images). The Fig. 1 shows a general diagram of granular computing to create a mosaic of images according to the approach described in the paper.

### Endoscope capsule

**Fig. 2:**
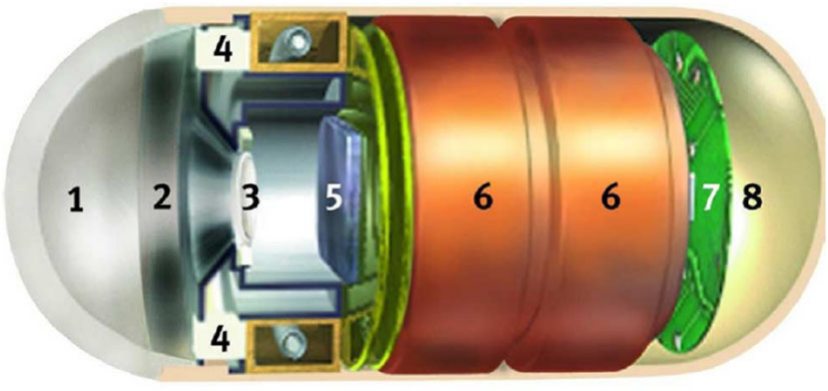
The layout of an endoscope capsule: *1* optical capsule, *2* lens holder, *3* lens, *4* light-emitting diodes, *5* system of CMOS image acquisition, *6* battery, *7* ASIC transmitter, *8* antenna

An endoscope capsule, which the study material comes from, is a modern approach while studying human gastrointestinal (Cunha et al. 2008). It enables to avoid gastroscopy—a very inconvenient examination as well as allows for multiple search of the whole human gastrointestinal since the images are saved on a computer disc. The layout of a typical endoscope capsule (Cunha et al. 2008) has been presented in Fig. 2.

The process of endoscope examination by means of the capsule goes as follows. First, an antenna device is placed on a patient’s belly making it possible to receive a visual signal transmitted by the capsule. A patient swallows an endoscope capsule which goes through the whole human gastrointestinal taking 2 images per second which gives according to the producer over 50,000 images while being examined. The capsule is disposable. The images sent to an external device are placed on disc of a computer with Rapid application which serves as a browser for the images.

### Image mosaicing and image registration

Image mosaicing (Yue et al. 2008; Kanazawaa and Kanatanib 2004) is a process involving creation of one image through bringing all images onto one surface, that is such laying of images coming from various camera positions or several cameras that they create one image including all images. To make it possible, the images must have one intersection or the epipolar geometry among the cameras must be known (calibrated cameras). A task that is similar to image mosaicing is image registration. The latter differs from the former that the images do not necessarily come from different camera positions, but they may be of different types and from different sources. In literature, the image registration is also meant as both image mosaicing and image registration. Plenty of techniques of image registration can be helpful in image mosaicing, yet mosaicing is often harder because of perspective transformation between the images. Due to the variety of images which are supposed to be laid, and because of other conditions, it is impossible to design a universal method of laying, nevertheless each technique consists of the following steps (Zitova and Flusser 2003).

1. *Feature extraction* The important and distinctive structures are extracted (areas of closed boundary, edges, outlines, intersection of lines, corners, etc.). For further processing these features can be represented by points (centers of gravity, line endings, distinctive points ) which, in literature, are called control points (see Zitova and Flusser 2003).
2. *Feature matching* A correspondence is found between the features in the processed image and the found features in the reference image. For this purpose, a variety of descriptors of features and similarity measures with the spatial relationships among features are used.
3. *Estimation of the transformation model* The type and model of the mapping function is determined between the overlaid image and the reference image. Mapping function parameters are calculated by using the fixed correspondence features.
4. *Resampling and image transformation* The relevant image is transformed by using the mapping function. The values in the image at the points of non-integer coordinates are converted by suitable interpolation technique.

As a result of the analysis of techniques found in literature concerning matching of keypoints, the only techniques based on descriptors Speeded Up Robust Features (SURF) (Bay et al. 2008) and Scale Invariant Feature Transform (SIFT) (Lowe 2004; Chen and Tian 2006) possess properties that enable them to fit the keypoints in the images from the endoscopy capsule, since they purely function independently of the rotation, changes in brightness and scale among the images. Other techniques may be used only in the case of knowing in advance the approximate transformation. As a result of preliminary studies involving the comparison of the operation techniques of matching SURF and SIFT, the images from the endoscopy capsule proved that the technique SIFT worked better than SURF. Therefore, the SIFT technique was selected for use in the initial stage of the developed algorithm.

Key points selection and 2 nearest neighbors matching technique comes from SIFT algorithm which is standard, most popular technique to matching images. However, a slight modified method of 2 nearest neighbors (finding of N best matches by sorting them in terms of distance to 2 nearest neighbors), comes from GDB-ICP algorithm, was used.

## The developed algorithm for image mosaicing

This section presents the results of research which lead to the creation of an original algorithm for mosaicing images from the endoscopy capsule, as well as a complete description of the algorithm. This algorithm is the result of synthesis techniques and algorithms described in literature and designed for its needs. It has been called “Quadruple keypoints matching and perspective transformation testing (QKMPTT)” algorithm (Maciura 2012). In this paper we present this algorithm as a kind of a granular computing.

### Pre-processing 

In the developed algorithm, the pre-processing of the input images (two initial granules) plays an important role. It allows at a further stage (i.e., during the isolation of keypoints) to select those items that will be more useful in the search for the correspondence pairs. The first step in image pre-processing is the detection of noise which should not be taken into account when identifying and matching of the keypoints, since these are relative to the content which is moving along the gastrointestinal tract. The detection of the noise involves threshold saturation of colour (S) channel in the HSV color space (Palus and Bereska 1995). What is used here, is the fact that the ingesta has got much lower saturation level in relation to the wall of the gastrointestinal tract.

The second stage of image pre-processing is to identify the areas where it is best to look for keypoints, that is the area of major edges and corners in the image having a high contrast change. These locations are determined by the calculation of the intensity measurement, which will then be threshold to obtain a binary image:

$$\begin{aligned} M(x,y)=A(x,y)+B(x,y) \end{aligned}$$

where:

$$\begin{aligned} A(x,y)&= \sum _{u=x-r}^{x+r}\sum _{v=y-r}^{y+r} I_x(u,v)^2\ \omega (u,v),\\ B(x,y)&= \sum _{u=x-r}^{x+r}\sum _{v=y-r}^{y+r} I_y(u,v)^2\ \omega (u,v). \end{aligned}$$

where $I_x(u,v)$ and $I_y(u,v)$ are partial derivatives in the square neighboarhood of point $(x,y)$ sized $(2r+1)$ in vertical and horizontal, $\omega (u,v)$ is Gaussian weighting function (see in Harris and Stephens 1988).

Finally, using logical operations on binary images a binary image is created, which pixels does not belong to the noise, and at the same time belongs to the main edges and corners. This image will be used for subsequent rejection of the keypoints that do not lie in the right areas.

### The keypoints of input images 

The next step of this algorithm is to extract the keypoints of the input images (a collection of keypoints for an image we call as a *KP granule*). As a result of comparison of the available algorithms in terms of their characteristics as well as preliminary studies comparing keypoints of registration techniques there was selected SIFT technique as the matching algorithm of single keypoints in the developed algorithm. Thanks to the SIFT technique we obtain for any keypoint a vector of qualities (features), called a vector of SIFT descriptors. Such vectors are computed on the basis of keypoints surrounding and are used for further calculations. Besides, thanks to it the KP granules are replaced by two VKP granules which are the collection of vectors calculated for all keypoints from equivalent KP granules. The next step was the removal of the keypoints (from KP granules and from VKP granules) that lie on the edge of the screen and the ones that lie in the regions identified as noise caused by gastric contents (which detection was described in 2.1).

At the same time, the points are discarded which do not lie in the areas around the major edges and corners, based on the binary image described previously.

### The technique for finding the four best correspondences 

The technique for finding the four best correspondences has been developed for this algorithm. It is one of its main components. It is based on the fact that all possible combinations of quadruple matches of $N$ best rated matching (granules of pairs of VKP granules) is compared (in terms of the ratio of distance of the matched descriptor to the two nearest neighbors—in the sense of similarity) and the best quadruples are found. Algorithm (and number) of finding $N$ best rated matching cames from initial step of generalized dual-bootstrap iterative closest points (GDB-ICP) algorithm (Yang et al. 2007; Yang 2007) and from own experiments.

If there are fewer than $N$ matches, all possible combinations of sets of quadruples of all matches are compared. The quadruples are evaluated using the special assessment function. The lower value of the evaluation function, the better the given quadruple. The evaluation function takes into account the differences in the SIFT descriptors and the geometric similarity of the matched quadruples. This function is described by the following equation:

1 $$\begin{aligned} F=\sum _{i=1}^{4}D(P_i,P_i') \ \sqrt{\sum _{k=1}^{12}(A_k-A_k')^2} \end{aligned}$$

where $D(P_i,P_i')$ is the distance of SIFT descriptors (on the account of their similarity) for 4 corresponding points $P_i$ and $P'_i$, and in both images, $A_k - A'_k$ are the differences of twelve corresponding to each other all possible angles formed out of four points in both images. Four correspondences is minimal number to estimate perspective transformation matrix (which is described in 2.5), and 12 is a number of all possible angles between keypoints in quad ($4 \cdot C^2_3 = 4 \cdot (^3_2) = 12$).

After sorting all quadruple combinations in terms of their evaluation function, among the top quadruples one should find the correct solution (if any). This technique works best with the assumption that the overall perspective transformation between the images is similar to the affine transformation.

### Setting the optional perspective transformations between images 

The next step is to determine different versions of perspective transformations between a pair of images. This is done repeatedly in the main loop of the program where different versions of the transformation based on the $M$ best SIFT correspondence quadruples are calculated. Finally, the best version of the transformation is considered valid and used to create the mosaic. Computation of the transformation is done using an algorithm determining the perspective transformation based on the correspondence of four points. Analyzing the perspective transformation matrix, it is possible to reject at this stage of transformation errors resulting from incorrect quadruple matches. One can check whether certain transformation parameters have the correct values. It significantly speeds up the functioning of the algorithm as incorrect transformations need no longer be analyzed and can proceed to the next iteration of the main loop.

### Estimation of perspective transformation matrix using four matched keypoints

This algorithm is implemented in *OpenCV* library in function *cvGetPerspectiveTransform*. The result of this algoritm is perspective transformation matrix $3 \times 3$ (Eq. 2). Four matched keypoints is minimal number to calculate perspective transformation matrix.

There are four points: $P_1=(x_1,y_1)$, $P_2=(x_2,y_2)$, $P_3=(x_3,y_3)$, $P_4=(x_4,y_4)$ and its corespondences (matched keypoints in a second image): $P'_1=(x'_1,y'_1)$, $P'_2=(x'_2,y'_2)$, $P'_3=(x'_3,y'_3)$, $P'_4=(x'_4,y'_4)$. Perspective transformation matrix H (Eq. 2) can be calculated by solution of following equation using singular value decomposition (SVD) technique.

$$\begin{aligned} \left[ \begin{array}{cccccccc} x_1 &{} y_1 &{} 1 &{} 0 &{} 0 &{} 0 &{} -x_1 x'_1 &{} -y_1 x'_1\\ x_2 &{} y_2 &{} 1 &{} 0 &{} 0 &{} 0 &{} -x_2 x'_2 &{} -y_2 x'_2\\ x_3 &{} y_3 &{} 1 &{} 0 &{} 0 &{} 0 &{} -x_3 x'_3 &{} -y_3 x'_3\\ x_4 &{} y_4 &{} 1 &{} 0 &{} 0 &{} 0 &{} -x_4 x'_4 &{} -y_4 x'_4\\ 0 &{} 0 &{} 0 &{} x_1 &{} y_1 &{} 1 &{} -x_1 y'_1 &{} -y_1 y'_1\\ 0 &{} 0 &{} 0 &{} x_2 &{} y_2 &{} 1 &{} -x_2 y'_2 &{} -y_2 y'_2\\ 0 &{} 0 &{} 0 &{} x_3 &{} y_3 &{} 1 &{} -x_3 y'_3 &{} -y_3 y'_3\\ 0 &{} 0 &{} 0 &{} x_4 &{} y_4 &{} 1 &{} -x_4 y'_4 &{} -y_4 y'_4\\ \end{array} \right] H= \left[ \begin{array}{c} x'_1\\ x'_2\\ x'_3\\ x'_4\\ y'_1\\ y'_2\\ y'_3\\ y'_4\\ \end{array} \right] \end{aligned}$$

where $H={[a\ b\ c\ d\ e\ f\ g\ h]}^T$ is the vector containing elements of the perspective transformation matrix $H$:

2 $$\begin{aligned} H=\left[ \begin{array}{ccc} a &{} b &{} c\\ d &{} e &{} f\\ g &{} h &{} 1 \end{array} \right] \end{aligned}$$

### Transformation of image for evaluation of transformation matrix 

In order to select the best transformation matrix, it is necessary to perform each transformation and its evaluation first. When the transformation in the form of transformation matrix is known, its performance is a simple problem. To perform this transformation, a transformation technique was used based on the perspective transformation matrix (Eq. 2), and a bilinear interpolation technique (Goshtasby 2005).

New position of each point can be calculated from transformation matrix using simple Eqs. 3 and 4. In order to eliminate non-integer position bilinear interpolation technique was used [see Goshtasby (2005) for more details].

3 $$\begin{aligned} X=\frac{a\ x + b\ y + c}{g\ x + h\ y + 1}\end{aligned}$$

4 $$\begin{aligned} Y=\frac{d\ x + e\ y + f}{g\ x + h\ y + 1} \end{aligned}$$

For the best $M$ quadruples in a loop there are transformations calculated, which are done if the transformation matrix is correct (the scale parameters $a$ and $e$ from transformation matrix shown in Eq. 2 must be greater than 0). Equation 5 describes the correct values of the scale parameters.

5 $$\begin{aligned} S=\left\{ \begin{array}{ll} (0,1), &{} \text{ when } \text{ the } \text{ scale } \text{ decreases }\\ 1, &{} \text{ the } \text{ scale } \text{ is } \text{ unchanged }\\ (1, +\infty ), &{} \text{ when } \text{ the } \text{ scale } \text{ increases } \end{array} \right. \end{aligned}$$

The transformation matrices done on such a basis are then subjected to further evaluation.

### Finding the best transformation 

The next step in the main loop of the program is the analysis of the performed transformations to select the matrix of the best evaluated transformation. This matrix will eventually be used to create a mosaic. The algorithm of the search for the best transformation is to match the edges between the transformed target image and the reference image and counting the points belonging to the edges that overlap in the common part of both images and have a similar orientation (angle of the inclination of the edge). The number of these points is the evaluation of the analyzed transformation. The edges of the images are extracted using the Canny edge detector (Canny 1986). In addition to the very edges of the target image and the reference image, one needs to calculate the orientation of the image points, which will be used later to compare the matched edges in terms of the difference in the angles of their orientation. The calculation of the orientation takes place after a previous calculation of partial derivatives of functions of image brightness. The original function serves to evaluate a transformation which calculates the number of overlapping edge points in both binary images which have similar orientation. The number of overlapping points is an evaluation of a transformation; the more numbers, the better transformation.

### Normalization of windows 

During the mosaicing process a very important element is to create suitable windows for the transformed image and the image of the outcome as a mosaic. It often happens, as a result of a transformation, that a transformed image and the mosaic are bigger than the input images and moved towards them. If the same windows were used to create the transformed image and the mosaic as in the input images, the outcome could not fit in the window. Apart from increasing the size of the window, one should also move the input images so that the outcome of the transformation does not exceed the size of the window from the left size or from the top. The very process of windows normalization starts after finding the best transformation. At the beginning of the process of window normalization, the algorithm using the formula for points belonging to the circle verifies which coordinates $P (x, y)$ lie on a circle border of the field of view image from the endoscope capsule (that is, the first parameter of the program). After determining the boundary points, the algorithm calculates their new coordinates in which they will find after the operation of transforming the image. These coordinates are calculated on the basis of the transformation matrix. The next step is to find the four most extreme points from the obtained set of new coordinates of boundary points. These are: a point located on the extreme left, right, up and down. With it, you can calculate the required size of the window, and the required vector of the shift of the images (so the resulting image does not exceed beyond the left and beyond the upper edge of the window).

### The final transformation and the creation of mosaic 

The best transformation matrix determined on the basis of suitability points in the images after normalization (2.8) is used for the final transformation of the standardized target image. To complete the transformation with the resampling, the function cvWarpPerspective from the OpenCV library was used. It uses the technique of transformation on the basis of the perspective transformation matrix and bilinear interpolation technique. The last step of creating the mosaic images is the so-called image fusion (resulting in final granule). On the whole, the concept of image fusion means appropriate connection of information with each other for two or more images. A broader concept of fusion covers the registration of images, and then connecting with each other corresponding pixels. The research has established the following image fusion algorithms (left to choose by the user): fusion by the average arithmetical value of the RGB channels, the image fusion technique for maximizing the value of the RGB channels, fusion by the technique of color mixing, fusion combined with a reduction of noise. The fusion by the average arithmetical value of the RGB channels deals with inserting in the output image pixels generated by calculating the arithmetic mean of corresponding RGB channels of corresponding pixels in the pairs of images. The fusion technique for maximizing the value of the RGB channels deals with inserting in the output image pixels generated by calculating the maximal corresponding to the RGB channels of corresponding pixels in a pair of images. The fusion by the technique of color mixing deals with inserting in the output image corresponding to the weighted average of the RGB channels corresponding to each pixel in a pair of images. The weighting factors of the weighted average are calculated based on the ratio of the distance of a pixel from the boundaries of the shared part of images together with each image. The result is a smooth transition from the shared part of images to particular images. The fusion combined with the removal of noise is to select for the output image the pixel with the corresponding pixels in the input images which has a higher level of color saturation (S channel in the HSV color space). This is due to the fact that the noise and the black background around the proper image has a much lower level of color saturation.

### A comprehensive presentation of the developed algorithm of image mosaicing

The proposed algorithm of image mosaicing called the “Quadruple keypoints matching and perspective transformation testing (QKMPTT)” algorithm is as follows:

1. Reading of the target image and the reference image (initial granules),
2. Appointment of the uncertain pieces of images,
3. Determination of the reference image edge using the Canny technique and removal of these that are caused by noise,
4. The calculation of the gradient orientation for the points in the reference image,
5. Designation of areas surrounding the dominant edges and corners,
6. Determination of SIFT keypoints (KP and VKP granules) in the areas identified in the previous step and simultaneously parts not belonging to the unsure parts of the image (determined in step two),
7. Finding matches (pairs of VKP granules) of the SIFT keypoints between images,
8. Sorting of matches in a non- decreasing order in terms of the quotient of the distance to their two nearest neighbors (which causes the lineup of matches from the best to the worst)
9. If there are fewer than $L$ matches, it is followed by the cease of the algorithm and a notice is displayed about an insufficient number of the found matches,
10. If there are more than $N$ matches, then there will be considered a subset of $N$ the best assessed matches from all sets of matches, otherwise a collection of all of the matches will be taken into account,
11. Finding the best of all possible combinations of an N set of quadruples of SIFT adjustments (determined in the previous step) and their evaluation,
12. Sorting of the adjustments of quadruples in terms of evaluation,
13. For the first $M$ quadruples (best in terms of assessment) :
  - Determination of the transformation matrix on the basis of quadruple matches,
  - If the approximate transformation matrix is incorrect, there is a move to the next iteration of the loop,
  - Perform the transformation in the target image,
  - Determination of the edge with the Canny technique for a transformed target image,
  - The calculation of the gradient orientation for points belonging to the target image after the transformation,
  - The calculation of the number of overlapping edge points with similar orientation,
  - If the assessment of the analyzed transformation is better than the current maximum rating, followed by the saving of the transformation matrix and keypoints by which it was determined (granule of mosaic). The maximum rating becomes the assessment of the analyzed transformation.
14. Final phase (when the maximum score is greater than 0):
  - Normalization of windows (windows transformation of the input image with their shift, and the creation of windows of appropriate size for a transformed image and the resulting image mosaic),
  - In case of the improper parameters of windows the program is stopped and the message comes out about the impossibility of the completion of the mosaics,
  - The creation of a new transformation matrix from the saved keypoints, if there has been the shift in images while normalizing the window,
  - Perform the transformation based on the best transformation matrix,
  - Completing the mosaic (final granule)
  Otherwise, the system displays an inability to create a mosaic.

## Experiments and results

The created “Quadruple keypoints matching and perspective transformation testing” algorithm is compared with the algorithm GDB-ICP proposed by Yang et al. (2007) in terms of accuracy and operating time. Both algorithms are completely different, although they are similar in initial stage, a technique for determining the N best correspondence SIFT was borrowed from GDB-ICP algorithm. Algorithm GDB-ICP consists in calculating the approximate transformation using single SIFT correspondence and then increase the accuracy of transformation estimation by matching points using another method and increasing the area around matching SIFT key points. In contrast created algorithm consists in calculating and testing perspective transforms using fours matched SIFT keypoints selected from set of N matched keypoints.

The algorithm GDB-ICP was the only one discovered by the author who handled the mosaicing images from endoscopy capsule and was available online in the form of an executable file. Unfortunately, the source of this algorithm was not available, so it was not possible to make any modifications. Publications Yang (2007) and Yang et al. (2007) describing the algorithm contained too little information would be possible to write a single source code. The disadvantage of the algorithm GDB-ICP is a very long computation time. Both the algorithm GDB-ICP and the developed QKMPTT algorithm downloaded two input images and the result was saved as a file. The table below shows the total results of the two algorithms for the tested pairs of images. These are qualitatively assessed correctness of the creation of mosaic and its creation time. The correctness is evaluated according to the following scale:

- +++—perfect or near-perfect mosaic,
- ++—visible mosaic comprising a transformation errors but quite correct,
- +—mosaic containing large errors in the transformation, but approximately correct,
- 0—mosaic totally incorrect or missing output file.

In Table 1 we give the results of experiments in applying the developed QKMPTT algorithm and the algorithm GDB-ICP.

The selected pairs of images come from different parts of the gastrointestinal tract. It is worth noticing that in the case of images from capsule endoscopy many images are such that even the human would not be able to match successive images (not to mention for algorithms working in the automatic way). On the other hand, in some places of the gastrointestinal tract capsule retracts or moves very slowly (which causes successive images with no difference). Therefore at this point we used the opinion of medical experts who helped point out some interesting seven pairs of images, which on one hand are significantly different from each other and on the other hand are representative examples of pairs of images whose can be matched automatically.

In the experiments the algorithm parameters $L$, $N$, $M$, was set to following values: $L = 10$, $N = 50$, $M = 100$. Number of minimal $L = 10$ found matches was determined from experimets consisting in testing numbers from $4$ to $50$ (with increment value $1$). Algorithm (and number) of finding $N = 50$ best rated matching comes from initial step of GDB-ICP algorithm and from own experiments. Number of $M = 100$ quadruples was determined from experiments consisting in testing numbers from $10$ to $200$ (with increment value $10$).

**Table 1:** Results of experiments with QKMPTT algorithm and the algorithm GDB-ICP

Pair	QKMPTT		Algorithm GDB-ICP	
	Accuracy	Time (s)	Accuracy	Time (s)
1	+++	6	+++	177
2	0	–	0	166
3	++	4	+++	232
4	+++	5	++	289
5	++	3	+	290
6	++	4	0	208
7	+++	7	+++	37

In conclusion, the discussed series of experiments of the designed “Quadruple keypoints matching and perspective transformation testing” algorithm in terms of accuracy works equally well as the algorithm GDB-ICP. Undoubtedly, the advantage of the designed algorithm is its speed. In all such cases, it operated faster than GDB-ICP algorithm. In the best case, 96.7 times faster and at worst 5.3 times faster than the algorithm GDB-ICP.

Execution time of both algorithms is given in seconds not in order to determine their complexity but in order to compare the speed of these algorithms. The algorithm GDB-ICP was available on the Internet in the form of exe file. On the basis of the publications about this algorithm it is also not possible to determine precisely its complexity. The described QKMPTT algorithm, which was compared with the algorithm GDB-ICP, uses a variety of image processing algorithms [for example the Canny edge detection (1986)], that the complexity of these algorithms is not clearly described in publications known from literature. Therefore, a formal computation of complexity of the QKMPTT algorithm seems to be very difficult or even impossible in this place.

**Fig. 3:**
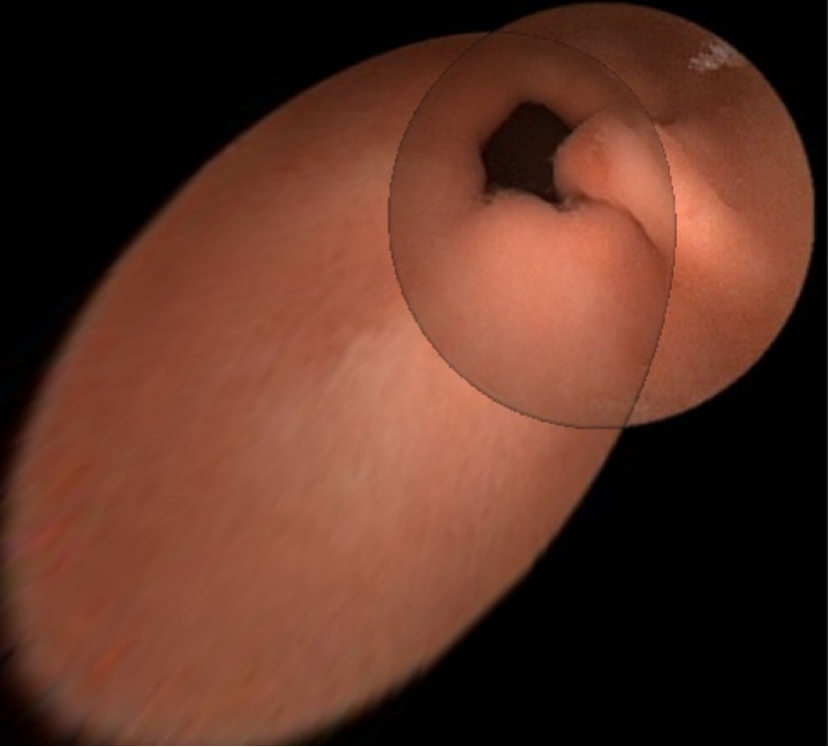
Mosaic created from pairs of images from the endoscope capsule. A fusion method was used by means of the arithmetic average of the RGB channels

**Fig. 4:**
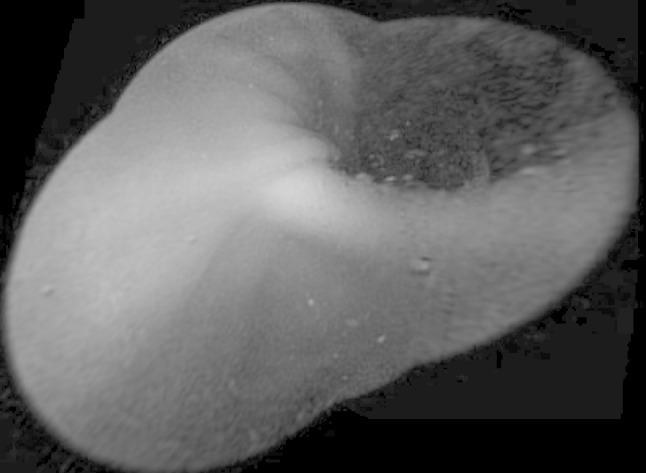
Mosaic created from three images from the endoscope capsule. A fusion of color mixing process was used here

The mosaic of a sample pair of endoscopy capsule images created using the developed Quadruple algorithm is presented in Fig. 3. The proposed algorithm can also create a mosaic of more images. For example, to create a mosaic out of three images you must first create a mosaic of a pair of images and the result of this mosaic should be specified as the second parameter in the next program call by joining another image to the mosaic as the first parameter. An example of this mosaic we can see in Fig. 4

## Conclusions

We have discussed methods for modeling of compound granules used in algorithms which could successfully construct a mosaic from the images coming from an endoscope capsule. The research was conducted on existing algorithms of applying and mosaicing of images, a selection of algorithms was improved and developed such algorithm which would be able to effectively construct a mosaic of images from the endoscope capsule. The presented algorithm is called “Quadruple keypoints matching and perspective transformation testing (QKMPTT)” algorithm. It has also been developed an algorithm to eliminate noise in the images of endoscopy capsule during the image fusion. After the final experimental studies, it turned out that the developed algorithm is many times faster than a commercial algorithm GBD-ICP for the images from endoscopy capsule and, at the same time, comparatively accurate. It should also be noted that the algorithm GDB-ICP was the only algorithm found by the author that handled the mosaicing of these images (other than the algorithm presented in this paper). The time in which the algorithm for images of the endoscopy capsule was developed gives hope for its implementation in real-time through the use of existing hardware capabilities (e.g., through parallelization of the algorithm using graphic processors with the CUDA technology). The parallelization of the algorithm would also open the possibility of adapting the algorithm to the application of registration endoscopy capsule images and CT images. The aim of the work presented in the article was not to create a diagnostic tool but only the tests that could make it possible to create such a tool. It should be noted that the research in this area and, in particular, the results obtained during the research are original. An expert judgment in this phase of research was limited to determining whether the obtained theoretical results promise the possibility of practical applications.
